# Noninvasive Self-diagnostic Device for Tear Collection and Glucose Measurement

**DOI:** 10.1038/s41598-019-41066-8

**Published:** 2019-03-20

**Authors:** Seung Ho Lee, Yong Chan Cho, Young Bin Choy

**Affiliations:** 1 Institute of Medical & Biological Engineering, Medical Research Center, Seoul National University, Seoul, Korea; 2 Interdisciplinary Program in Bioengineering, College of Engineering, Seoul National University, Seoul, Korea; 3 Department of Biomedical Engineering, Seoul National University College of Medicine, Seoul, Korea

**Keywords:** Diabetes, Diagnosis

## Abstract

We propose a noninvasive, self-diagnostic device that enables safe tear collection and glucose measurement. The device described herein was manufactured by tight assembly of a lid for tear collection in conjunction with a strip-type glucose sensor. The lid was designed to be in contact with the inferior palpebral conjunctiva for tear collection and was thus designed to possess a proper contact area and rounded boundaries to avoid eye tissue damage. For the strip-type glucose sensor, we employed a commercially available electrochemical sensor (Accu-Chek test strips), which was modified to reduce the volume of the reaction chamber (0.4 μl) for a small amount of collected tear fluid. When tested with *in vivo* animal models, the device was able to collect tear fluid in a relatively short time (<2 s) without causing eye tissue damage, and the device allowed the collected tear fluid to be delivered to the sensor for measurement of tear glucose concentrations. The blood glucose concentrations estimated with the tear glucose concentrations obtained with the device exhibited a high correlation with those actually measured with a clinically available glucometer (R^2^ = 0.9617).

## Introduction

Diabetes has been reported to be largely influenced by genetic factors^1,2^ and thus, early diagnosis of prediabetes, followed by proper treatment, has been shown to prevent progression to Type 2 diabetes, hence improving the quality of patients’ life^3,4^. In this sense, albeit healthy, the people with a high chance of getting diabetes can benefit from watching the blood glucose level early; however, with the currently-available strategies, blood needs to be withdrawn by pricking the fingertip with a needle for self-assessment of blood glucose^5,6^. This relatively invasive, painful measurement process often results in discomfort, even causing massive callous formation or loss of sensibility at the site of multiple punctures^7^. Therefore, this was considered one of the major reasons leading to an insufficient number of blood glucose tests even for diabetic patients^8^.

Therefore, much effort has been devoted to developing a less invasive, more convenient strategy for glucose measurement^9,10^. Among them, glucose measurement through tear fluid has drawn a great deal of interest. The glucose level in tears is known to have a demonstrable correlation with that in blood^11^, and tears can be collected relatively easily from the anterior space of the eye, which can be less invasive compared with conventional blood withdrawal through skin punctures. Therefore, many different measurement strategies, such as biological^12^, electrochemical^13,14^, spectroscopic^15,16^ and colorimetric^17^ measurements, have been extensively investigated for a more accurate measurement of glucose concentrations in tears.

However, those studies have not yet fully considered a practical methodology in the context of self-diagnosis. First, the two-step measurement procedure is not convenient for patients: tear fluid is collected separately and is then transferred and loaded to the analytical instrument for measurement. A relatively large volume of approximately 100 μl of tear fluid is often collected, which requires a long time of tear collection, as only approximately 6.5 μl of tear fluid is available at the preocular surface and the average rate of tear production is very low (1 μl min^−1^)^18,19^. Moreover, tear fluid may be collected using a glass capillary or water-absorbing paper; however, this technique has not yet been fully proven to be appropriate due to its invasiveness to the eye when used by the patients themselves. Therefore, these conventional strategies can still cause uncomfortable eye irritation or even damage and this can also lead to inaccurate measurements due to reflex tear production or an irrelevant increase in tear glucose due to the damaged tissue in the anterior surface of the eye^8,20–23^.

Therefore, in this work, we propose a device for glucose measurement in tears (i.e., a tear-glucose device), meeting the following design criteria: for the convenience of the patients, the tear-glucose device needs to be equipped with both a tear-collector and a sensor as a single entity, thereby allowing for concurrent collection and measurement of tear fluids. In this device, a tip needs to be designed minimally invasive as it must be in contact with the preocular surface (i.e., an ocular tip). Tear collection should be conducted for a short time, hence resulting in a small volume of collected tear fluid. The sensor also needs to be sensitive enough to accurately measure glucose concentrations in a small quantity of tears. At the same time, we also proposed a concept of device design that could be quick to apply to clinical applications and easy to manufacture.

To prepare such a tear-glucose device, we prepared an assembled entity consisting of a lid and a strip-type glucose sensor. We engineered the lid to consist of a minimally invasive ocular tip and a hollow body with a slit, where the strip-type glucose sensor could be inserted for tight assembly. In the ocular tip, a micron-size inlet was prepared, where after its contact with the preocular surface, a tear could be absorbed and delivered to reach the sensor inserted in the lid. For the strip-type glucose sensor, we employed a commercially-available test strip (Accu-Chek Performa, Roche Diagnostics, Switzerland), which is known to measure glucose levels via electrochemical means with high accuracy and selectivity^24^. However, in this work, we modified this test strip to be suitable specifically for the collection and measurement of a small quantity of tear fluid. Therefore, the lid herein possessing a simple structure would not be difficult to manufacture. The glucose test strip has been already widely used in clinical settings and thus, its manufacturing process is already well developed for a large-scale production.

The tear-glucose device herein was first tested at the range of physiological glucose concentrations present in tears under *in vitro* simulated environments. To test *in vivo* usability, the device was placed in contact with the inferior palpebral conjunctiva (IPC) of rabbit eyes to collect and measure tear fluids, since the IPC can be considered less sensitive compared with other regions in the preocular space^25^. We also validated the correlation between glucose concentrations measured in blood and tears. After application of our device, the IPC tissue was also examined for the purpose of safety evaluation.

## Results

### Lid

**Figure 1:**
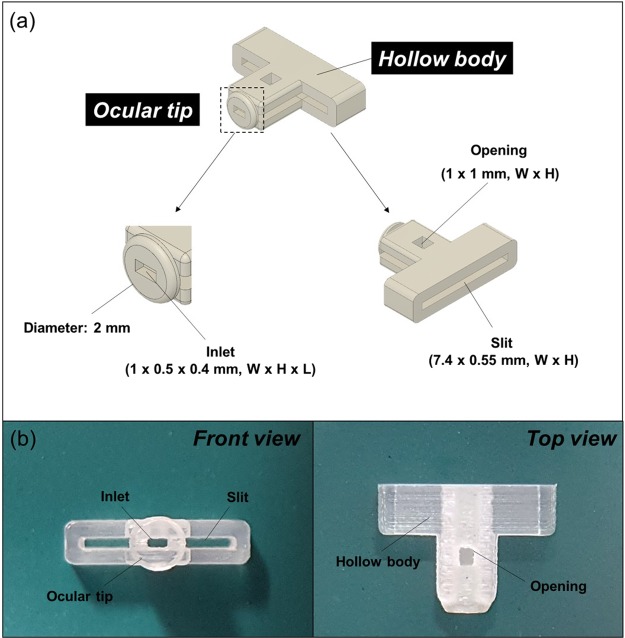
Lid for noninvasive tear collection. (**a**) Schematic images and (**b**) optical images of the lid prepared in this work.

We prepared the lid to serve as an interface between the eye surface and glucose sensor to safely collect and deliver tear fluids. To achieve this, we first designed the lid to consist of two distinct parts: the ocular tip and the hollow body using SolidWorks (Dassault Système SOLIDWORKS Corp., USA). As shown in Fig. 1(a), the ocular tip was shaped to be circular (2 mm in diameter) with rounded boundaries, which has been reported to not damage the IPC tissue during contact according to a previous study conducted by our group^26^. In the ocular tip, a small inlet (1 × 0.5 × 0.4 mm, W × H × L) was prepared to be able to absorb tear fluids via capillary action after contact with the IPC and to minimize the dead volume of tear fluid trapped inside of the inlet. The hollow body was designed to possess a slit (7.4 × 0.55 mm, W × H) for insertion of the strip-type glucose sensor used herein. The opening (1 × 1 mm, W × H) was also made at the top of the hollow body so that tear fluids could be smoothly absorbed and transferred to the glucose sensor. Figure 1(b) shows the lid fabricated with a 3D printer (ProJet 3500 HD, 3D Systems, USA), using the printing material Visijet M3 Crystal, certified by USP (United States Pharmacopeia) Class VI.

### Strip-type glucose sensor

To prepare a strip-type sensor, we employed the commercially available Accu-Chek test strip and modified it for the purpose of glucose measurement in tears. This test strip was equipped with an electrochemical sensor with a low limit of quantification (<45 μM) and high selectivity for glucose measurement^27^. In this sensor, collecting a fixed volume of fluid sample is important for accurate measurement of glucose, as the electrical current that varies according to the rate of glucose oxidation is also influenced by the volume of fluid in the reaction chamber. The Accu-Chek test strip is originally designed to measure glucose levels in the blood, and thus the reaction chamber with the electrodes is shaped to hold 0.8 μl of fluid.

**Figure 2:**
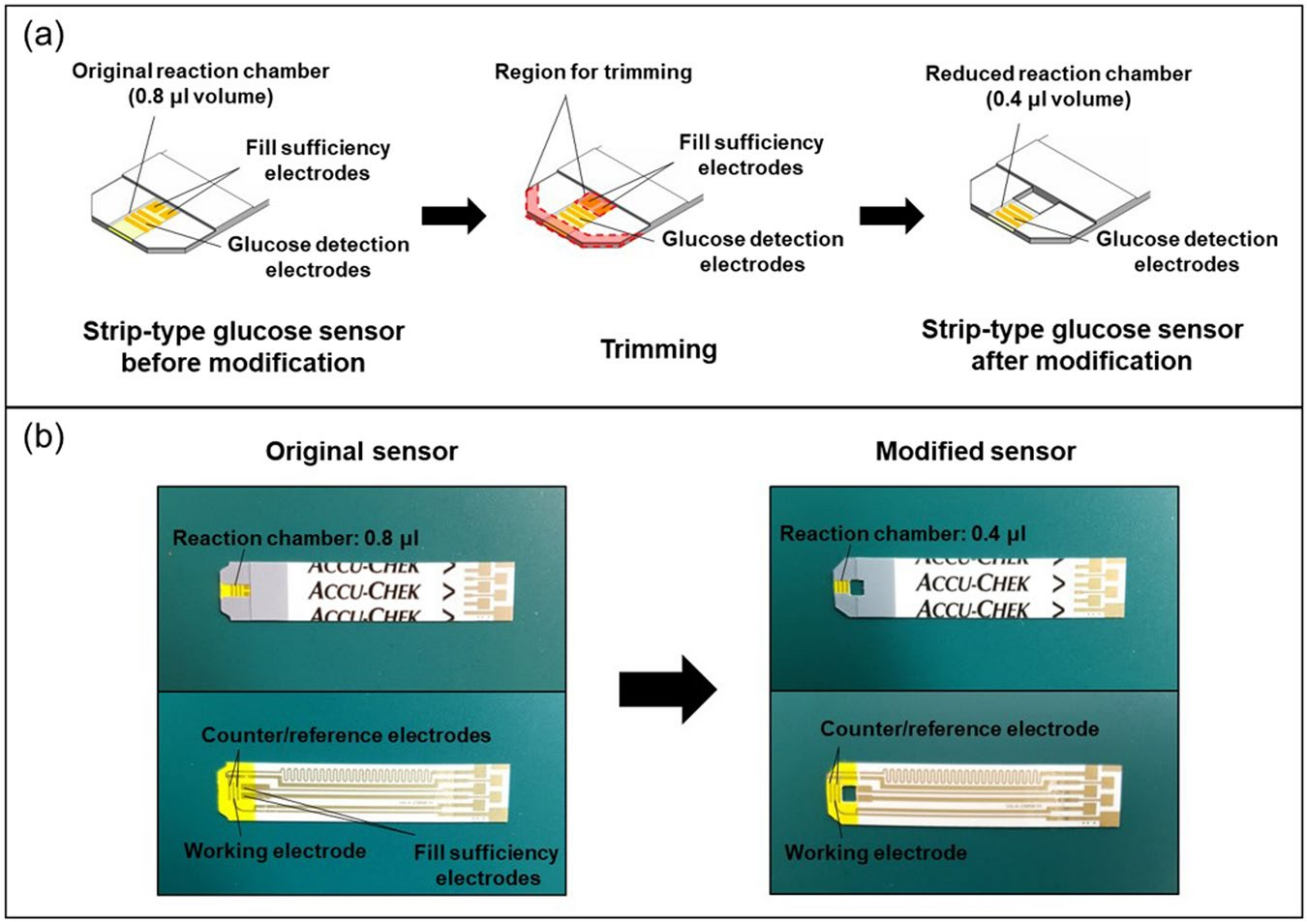
Modification of strip-type glucose sensors. (**a**) Schematic description of sensor modifications to reduce the volume of the reaction chamber. (**b**) Optical images of the strip-type glucose sensors before and after modification, showing the embedded electrodes and circuits.

In this work, we attempted to minimize the volume of collected tear fluid, so we modified the test trip mainly to decrease the volume of the reaction chamber. During this process, the major electrodes for glucose measurement needed to be kept intact. In the reaction chamber of the Accu-Chek test strip, four different electrodes were embedded; the counter, reference, working and fill-sufficiency electrodes. For glucose measurement, the counter, reference and working electrodes must be kept intact. However, the fill-sufficiency electrodes for detection of the presence of liquid might not be necessary to test the prototype device prepared in this work. Given these facts, as shown in Fig. 2, we first cut out the frontal end of the strip to the extent where the counter, reference and working electrodes were not affected, to reduce the length of the reaction chamber. We also rounded off the sharp edges in the frontal end to avoid possible damage to the IPC tissue. Then, we punched out the other end of the reaction chamber, removing the fill-sufficiency electrodes, to further reduce its volume. Thus, after modification, the resulting strip-type glucose sensor became equipped with a reaction chamber containing a volume reduced by half to 0.4 μl.

### Tear-glucose devices

**Figure 3:**
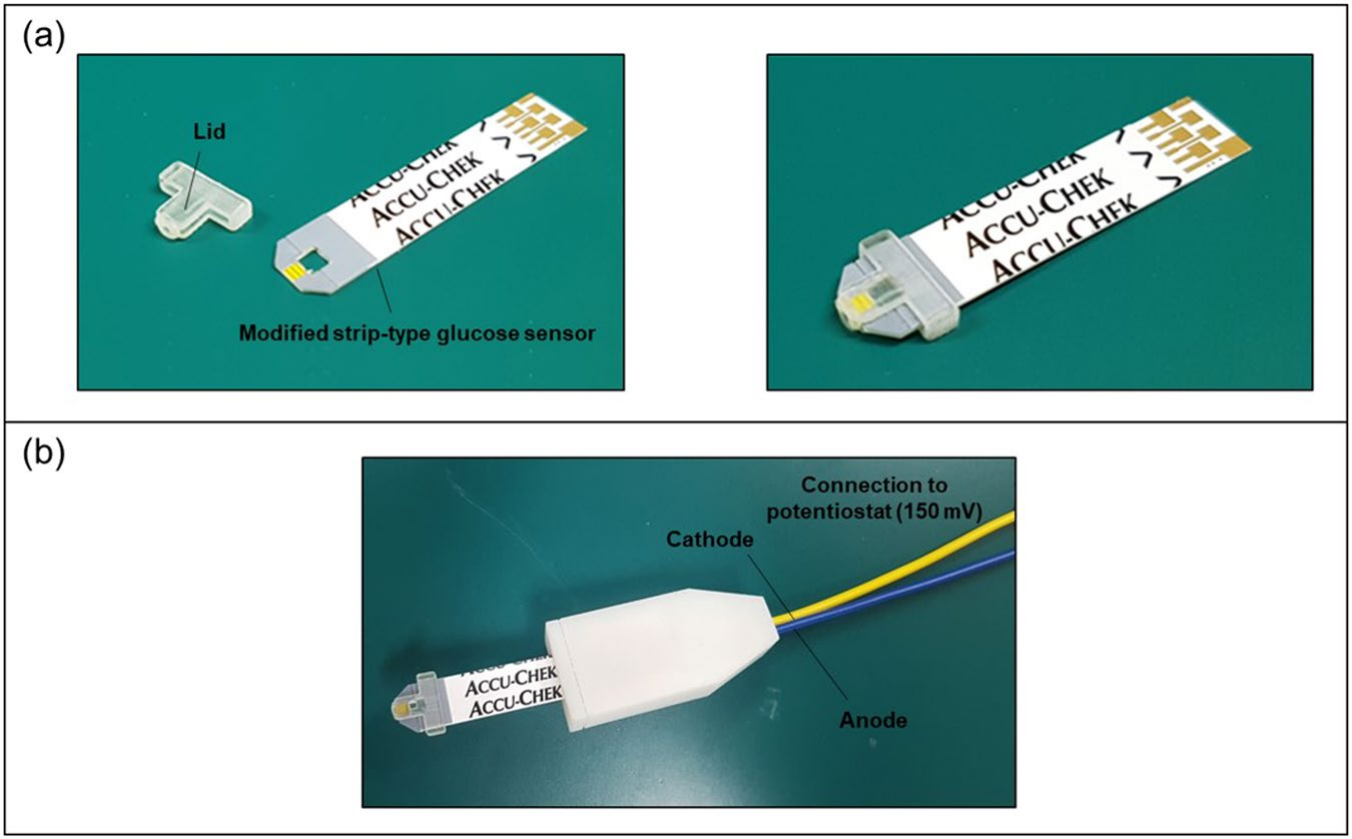
Preparation of the tear-glucose device. (**a**) Assembly of the lid and-modified strip-type glucose sensor. (**b**) Tear-glucose device connected to the potentiostat. The counter and reference electrodes were connected together as a single cathode and the working electrode worked as an anode.

The tear-glucose device herein described was prepared by simple assembly of the lid and the modified strip-type glucose sensor, as shown in Fig. 3. After insertion in the slit, the strip-type sensor was tightly fit, and the reaction chamber was properly aligned to the inlet prepared in the ocular tip of the lid. After that, the counter, reference and working electrodes were each connected to the counterpart connection in a potentiostat to apply 150 mV of DC voltage and measure the electrical current. In this work, we used a two-electrode system and thus, the counter and reference electrodes were connected as a single cathode while the working electrode was connected separately as an anode (Fig. 3).

**Figure 4:**
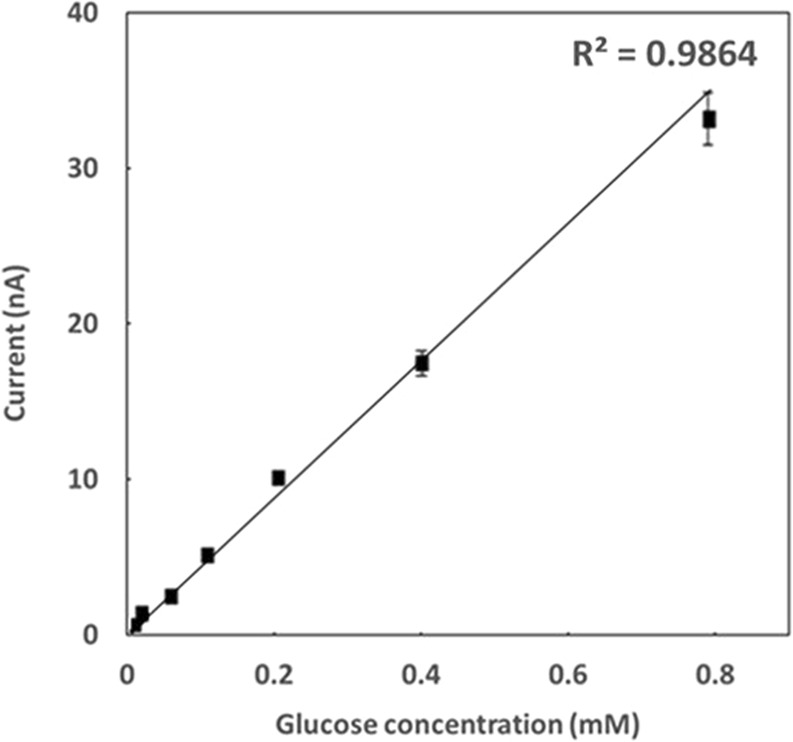
Calibration curve for varying glucose concentrations obtained with the tear-glucose device. The glucose solution was prepared by dissolving D-(+)-Glucose (Sigma-Aldrich, USA) at the concentrations of 0, 0.01, 0.05, 0.1, 0.2, 0.4 and 0.8 mM in phosphate-buffered saline (PBS) at pH 7.6, respectively. To collect 0.4 μl of the glucose solution, the ocular tip of the device was placed in contact with the glucose solution for 2 s. For measurement of the concentration of glucose, the tear-glucose device was connected to a potentiostat (DY2113, Digi-Ivy, USA) to apply 150 mV and the output current was obtained using the amperometric i-t curve mode.

To test the feasibility of the tear-glucose device herein, *in vitro* calibration was conducted with a glucose solution prepared in phosphate-buffered saline (PBS) with varying concentrations of 0, 0.01, 0.05, 0.1, 0.2, 0.4 and 0.8 mM. As shown in Fig. 4, a highly linear relationship was obtained between the glucose concentration and electrical current (R^2^ > 0.9864), indicating that the tear-glucose device herein described was valid to measure the *in vivo* glucose concentration in tear fluid, which was reported to range from 0~0.6 mM^28^.

### *In vivo* evaluation

To evaluate the feasibility of the tear-glucose device herein described, *in vivo* experiments were conducted using rabbits, where the blood glucose concentration was elevated through anesthetization^13,14,28^. In this work, the tear glucose concentrations were measured with the tear-glucose device and the blood glucose concentrations were measured with a blood glucometer approved for clinical use (Accu-Chek Performa, Roche Diagnostics, Switzerland) at scheduled times of 15, 30, 45, and 60 min after anesthetization.

**Figure 5:**
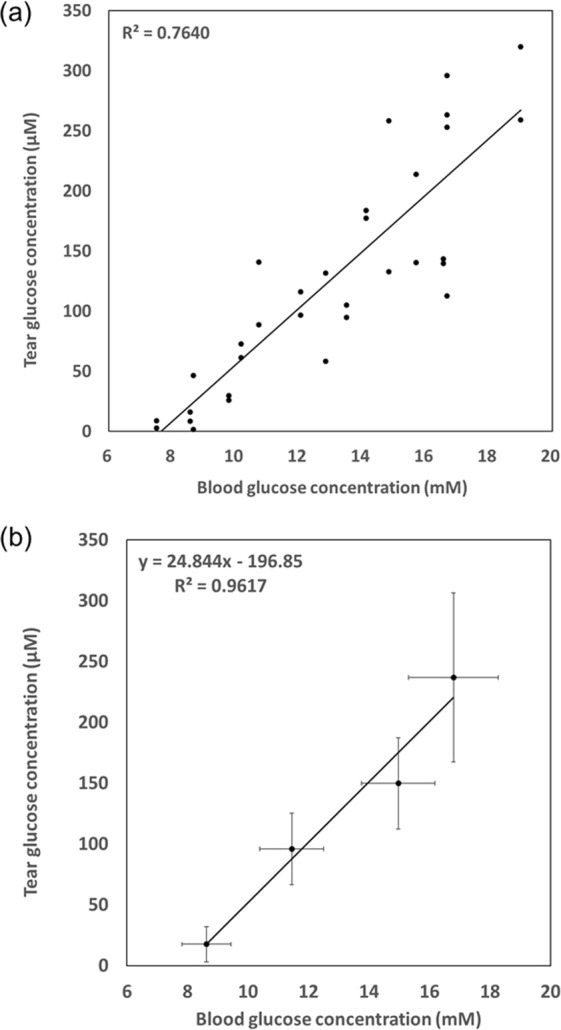
Correlation between blood glucose concentrations and tear glucose concentrations measured with the tear-glucose device. (**a**) Plots obtained with all eight rabbits (i.e., eight eyes) employed in this work. (**b**) Plots with average glucose concentrations in tear and blood obtained at each scheduled time of measurements.

Figure 5(a) shows the plot of the tear and blood glucose concentrations obtained from all eight rabbits tested in this work, showing a linear relation between tear and blood glucose concentrations. However, the data points were spread to some extent and hence the linear correlation appeared not to be very strong (R^2^ = 0.7640, df = 30, P < 0.05). This discrepancy was also observed in previous reports and was ascribed to the inherent differences among tested animals^13,14^. Therefore, when the data points averaged at each time of measurement (i.e., at every 15 min) were plotted^13^, as shown in Fig. 5(b), a strong linear correlation with a statistical significance was obtained (R^2^ = 0.9617, df = 2, P < 0.05).

**Figure 6:**
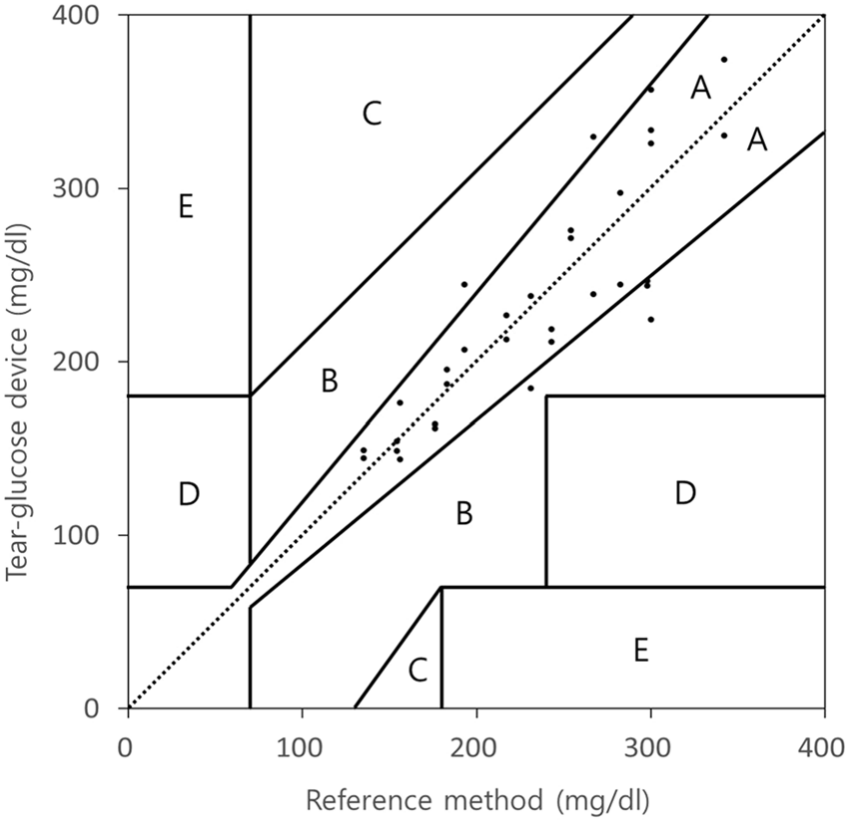
Clarke error grid analysis on blood glucose concentrations obtained with the tear-glucose device and a clinically approved glucometer. All data were plotted within zones A and B, showing the reliability of the blood glucose concentrations estimated with the tear-glucose device^29,30^. The y-axis represents the blood glucose concentration estimated from tear glucose concentration measured with the tear-glucose device, and the x-axis represents the blood glucose concentration measured with a reference method using a commercially available glucometer. Region A indicates that the data are within 20% error from the reference method. Region B indicates that the values are over the 20% error mark, but do not imply an inappropriate treatment. Region C indicates that the data will lead to an unnecessary treatment. Region D indicates that the data will fail to diagnose hyperglycemia or hypoglycemia. Region E indicates that the data will lead to a treatment for hypoglycemia to a patient with hyperglycemia, or vice versa.

In this study, we sought to show that our tear-glucose device herein described can estimate the blood glucose concentration based on the glucose concentration measured in tears. To evaluate this, the estimated and actual blood glucose concentrations were plotted in a Clarke error grid^29,30^. The estimated blood glucose concentrations were calculated from the tear glucose concentrations using the equation obtained from the graph in Fig. 5(b). The actual blood glucose concentrations were measured from the blood using a glucometer approved for clinical use. As shown in Fig. 6, all data points are in the zones A and B, suggesting that the blood glucose concentrations estimated with the tear-glucose device herein are highly correlated and possibly interchangeable with those conducted with the conventional blood glucose measurement method. Approximately 81.2% of the data points are in the zone A, implying a relatively higher accuracy of blood glucose measurement with the tear-glucose device described herein.

**Figure 7:**
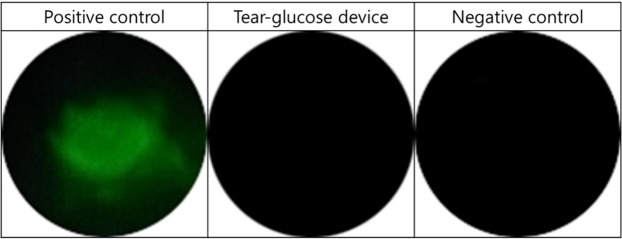
Representative fluorescent images obtained from the IPCs of fluorescein-stained rabbit eye. For the positive control group, a glass capillary tube was placed in contact with the IPC once. For the tear-glucose device group, the tear-glucose device was applied to the IPC four times at intervals of 15 min during 60 min. For the negative control group, no treatment was applied to the IPC.

To evaluate the safety of the tear-glucose device, we also examined the IPC tissues at the end point of the experiments, i.e., after each of the four times of contact of the tear-glucose device on the IPC, where the eye surface was stained with a fluorescein solution to assess possible tissue damage^31^. As shown in Fig. 7, after contact of the tear-glucose device, the IPC tissues were not observed to be stained, as seen with the nontreated, negative control eye, indicating no epithelial tissue damage. In contrast, when the IPC was in contact with a glass capillary tube, conventionally used for tear collection, an apparent sign of staining was observed due to damage of the IPC tissue.

## Discussion

Tear glucose measurement has been suggested as a potential, noninvasive strategy of blood glucose prediction^8,10,32^. Most of the previous studies focused on developing sensors with a higher accuracy since the glucose concentration in tears is known to be lower than that in the blood^13,33,34^. However, to our knowledge, studies on devices for practical, self-diagnostic applications is scarce. In this context, a device allowing concurrent tear collection and glucose measurement could be useful and convenient for users. Such a device would be more advantageous if the measurement could be reliable even with a small quantity of tear fluid as this would allow for a short time of tear collection, hence less invasiveness on the preocular tissues.

Therefore, we proposed the tear-glucose device herein as a noninvasive self-diagnostic tool for prediction of blood glucose levels. The lid in the device was shown to not damage the eye tissues due to its design with a proper contact area and rounded boundaries without sharp edges (Fig. 7)^26^. Although the lid herein was made of a slightly hydrophobic material (Visijet M3 Crystal, 3D Systems, USA), the micron size inlet still allowed for a strong capillary pressure, hence safe and efficient absorption of tear fluid, which could be rapidly transferred to fill the reaction chamber with a reduced volume (0.4 µl) in the strip-type glucose sensor (Fig. 2). With a lid made of a more hydrophilic material, it is expected that a more amount of tear can be collected in a more efficient manner^35^. Since the lid for tear collection and the strip-type sensor for measurement were assembled as a combined entity, the glucose concentration could be measured almost instantaneously after tear collection without its evaporation. Therefore, the whole process from tear collection to measurement took less than 2 s in this work. This short time of preocular contact would minimize eye irritation and possible measurement errors caused by reflex tear generation^8,13,23^. For measurement, we applied 150 mV to induce an electric current at 300 nA. Considering the maximum preocular contact time of 2 s, this would give at most 110 nJ, which is far less than that known to be safe when applied to the ocular tissues (12 J)^36,37^.

Our *in vivo* findings revealed that the glucose concentrations in tears measured with the tear-glucose device were highly correlated with those in blood measured with a glucometer in clinical use Fig. 5(a). This correlation was more prominent when the relations were plotted using the average glucose concentrations obtained at each measurement time Fig. 5(b). Those results suggested that the tear-glucose device herein could have a similar level of accuracy compared to a conventional glucometer (Fig. 6) and compared to the strategies reported in previous studies^13,14^. However, due to an inherent variation among individuals, as well as the presence of temporal correlation between the blood and tear glucose levels (Supplementary Fig. 1), there could be some limitations for treating accurate glycaemia events with the diabetic patients. In this sense, our device could be better suited for healthcare purposes, especially for early diagnosis of prediabetes. With ease of use and noninvasiveness, our device herein could be more favored by the people with a high chance of getting diabetes, allowing for watching the blood glucose level early without much of discomfort even under a healthy condition. Considering actual applications in human clinical settings, a strip-type electrochemical sensor with a higher sensitivity may need to be developed. For humans, the tear glucose concentrations may vary according to the tear collection methods, but are approximately 50 times lower than those in blood^38,39^ and this dilution factor was lower than that reported in rabbits (25 times)^28^. However, the device herein was able to measure the glucose concentrations at such lowered ranges expected in human tear fluids (Supplementary Fig. 2).

In conclusion, we propose a noninvasive self-diagnostic tear-glucose device that can perform rapid and concurrent tear collection and glucose measurement. The tear glucose concentrations can be measured with an electrochemical, strip-type sensor embedded in the tear-glucose device, which can estimate the blood glucose concentrations that were shown to be highly correlated with those measured with a conventional glucometer in clinical use. The proposed lid in the tear-glucose device can avoid eye tissue damage after contact with the inferior palpebral conjunctiva, hence minimizing irritation and the production of reflex tear fluids. Therefore, we envision that the tear-glucose device, a combined entity consisting of a lid and a sensor, has the potential to predict blood glucose levels, thereby allowing for early diagnosis of prediabetes.

## Methods

### *In vivo* experiments

Approval for the *in vivo* experiments was granted by the Institutional Animal Care and Use Committee (IACUC No. 15-0285) at the Biomedical Research Institute of the Seoul National University Hospital. We confirmed that all experiments were performed in accordance with relevant guidelines and regulations. Male New Zealand white rabbits (2.5~3.0 kg) were raised in a controlled environment (temperature: 21 ± 1 °C, humidity: 55 ± 1%, light/dark cycle: 12 hours, and food and water ad libitum). Hyperglycemia was induced in animal models via anesthetization, as reported in previous studies^13,14,28^. To achieve this, the rabbits were first anesthetized with a subcutaneous injection of a cocktail of 15 mg kg^−1^ ketamine (Ketamine: Yuhan, Korea) and 5 mg kg^−1^ xylazine (Rompun: Bayer, Germany) and after 40 min, a booster shot of 7.5 mg kg^−1^ ketamine and 2.5 mg kg^−1^ xylazine was given. The glucose concentrations in both blood and tears were measured at 15, 30, 45, and 60 min after the first shot of the anesthetics. To measure blood glucose concentrations, blood was drawn from the right ear vein of the rabbit using a lancet, and a glucometer was used (Accu-Chek Performa, Roche Diagnostics, Switzerland). To measure the tear glucose concentrations, the lid of the tear-glucose device connected to a potentiostat was in contact with the IPC of the rabbit eyes for 2 s. At the specified endpoints of the experiments, we examined the presence of tissue damage on the IPC surface, following a previously reported protocol^31^. Briefly, a 5-μl drop of 0.25% w v^−1^ fluorescein sodium solution was instilled in the eye and after 5 min, the eye was washed thoroughly with normal saline to remove excess fluorescein solution. Then, a fluorescent image of the IPC surface was obtained, using a camera (Galaxy S7, Samsung, Korea) equipped with excitation (475 nm) and emission (542 nm) light filters (Thorlabs, USA).

### Statistical analysis

Statistical analysis was performed with two-tailed Pearson’s correlations using SPSS (SPSS version 23, IBM, USA). A Student’s t-test was performed to obtain the P values. P < 0.05 was considered a statistically significant difference.

## Supplementary information

Supplementary information
