# Author Correction: Noninvasive Self-diagnostic Device for Tear Collection and Glucose Measurement

**DOI:** 10.1038/s41598-019-49489-z

**Published:** 2019-09-03

**Authors:** Seung Ho Lee, Yong Chan Cho, Young Bin Choy

**Affiliations:** 1 Institute of Medical & Biological Engineering, Medical Research Center, Seoul National University, Seoul, Korea; 2 Interdisciplinary Program in Bioengineering, College of Engineering, Seoul National University, Seoul, Korea; 3 Department of Biomedical Engineering, Seoul National University College of Medicine, Seoul, Korea

Correction to: *Scientific Reports* 10.1038/s41598-019-41066-8, published online 18 March 2019

This Article contains an error.

The Authors missed out a previous study on a similar topic. The additional reference is listed below as reference 1, and should appear in the text as below.

In the Discussion section,

“Tear glucose measurement has been suggested as a potential, noninvasive strategy of blood glucose prediction^8,10,32^. Most of the previous studies focused on developing sensors with a higher accuracy since the glucose concentration in tears is known to be lower than that in the blood^13,33,34^. However, to our knowledge, studies on devices for practical, self-diagnostic applications is scarce. In this context, a device allowing concurrent tear collection and glucose measurement could be useful and convenient for users. Such a device would be more advantageous if the measurement could be reliable even with a small quantity of tear fluid as this would allow for a short time of tear collection, hence less invasiveness on the preocular tissues.

Therefore, we proposed the tear-glucose device herein as a noninvasive self-diagnostic tool for prediction of blood glucose levels.”

should read:

“Tear glucose measurement has been suggested as a potential, noninvasive strategy of blood glucose prediction^8,10,32^. Most of the previous studies focused on developing sensors with a higher accuracy since the glucose concentration in tears is known to be lower than that in the blood^13,33,34^. A previous paper by Kownacka *et al*.^1^ reported results of phase II clinical trial for a device for continuous glucose monitoring in tear fluid, which needs to reside at the preocular surface while being wired to the reader. However, a device allowing sampled tear collection and glucose measurement could be also useful and probably more convenient for users. Such a device would be more advantageous if the measurement could be reliable even with a small quantity of tear fluid as this would allow for a short time of device contact for tear collection, hence less invasiveness on the preocular tissues.

Therefore, we proposed the tear-glucose device herein as an alternative noninvasive self-diagnostic tool for prediction of blood glucose levels.”
