# Supplementary material for: Noninvasive Self-diagnostic Device for Tear Collection and Glucose Measurement
Source: Sci Rep. 2019 Mar 20;9:4747. doi: 10.1038/s41598-019-41066-8 (PMC6427043; doi:10.1038/s41598-019-41066-8)
Supplement: Supplementary file 1 — Supplementary information [file 41598_2019_41066_MOESM1_ESM.docx]

**Noninvasive Self-diagnostic Device for Tear Collection and Glucose Measurement**

Seung Ho Lee^+^, Yong Chan Cho^+^, Young Bin Choy^*^

^+^ These authors contributed equally as first authors to this work.

* To whom all correspondence should be addressed

E-mail: [ybchoy@snu.ac.kr](mailto:ybchoy@snu.ac.kr)


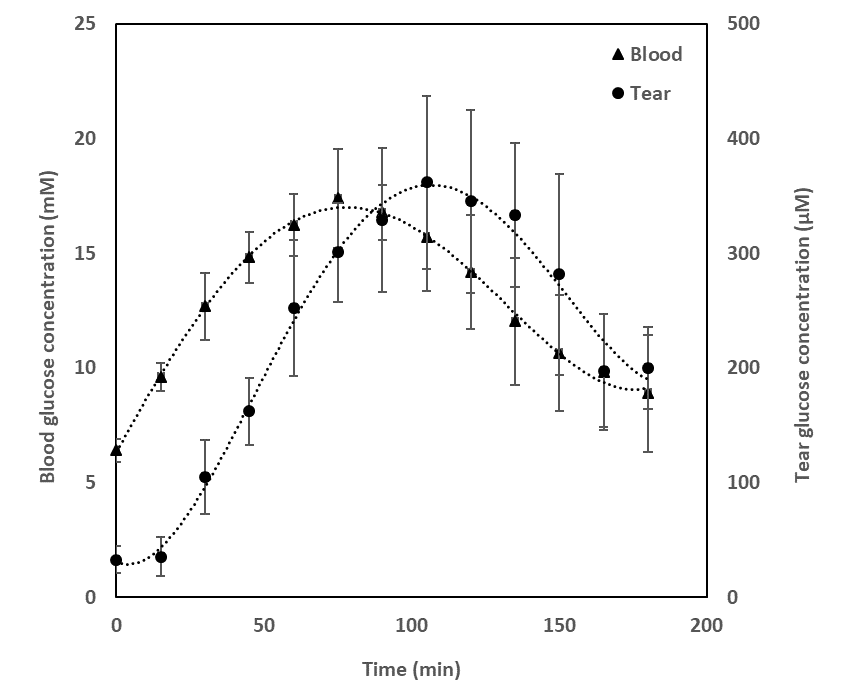


**Supplementary Figure 1. Temporal correlation between blood and tear glucose concentrations**, which were measured by a glucometer (Accu-Chek Performa, Roche Diagnostics, Switzerland) and tear-glucose device herein, respectively. To increase the blood glucose level, rabbits (n = 6) were anesthetized with a single, subcutaneous injection of a cocktail of 15 mg kg^-1^ ketamine and 5 mg kg^-1^ xylazine and they were left without boosters to decrease the blood glucose level afterwards. Both blood and tear glucose concentrations were measured every fifteen minutes after anesthetization. Our results revealed that a lag time was indeed present between the profiles of blood and tear glucose concentrations, as reported in previous studies [^1^](#_ENREF_1)^,^[^2^](#_ENREF_2), which implied an acceptable accuracy of the tear-glucose device prepared in this work.

**
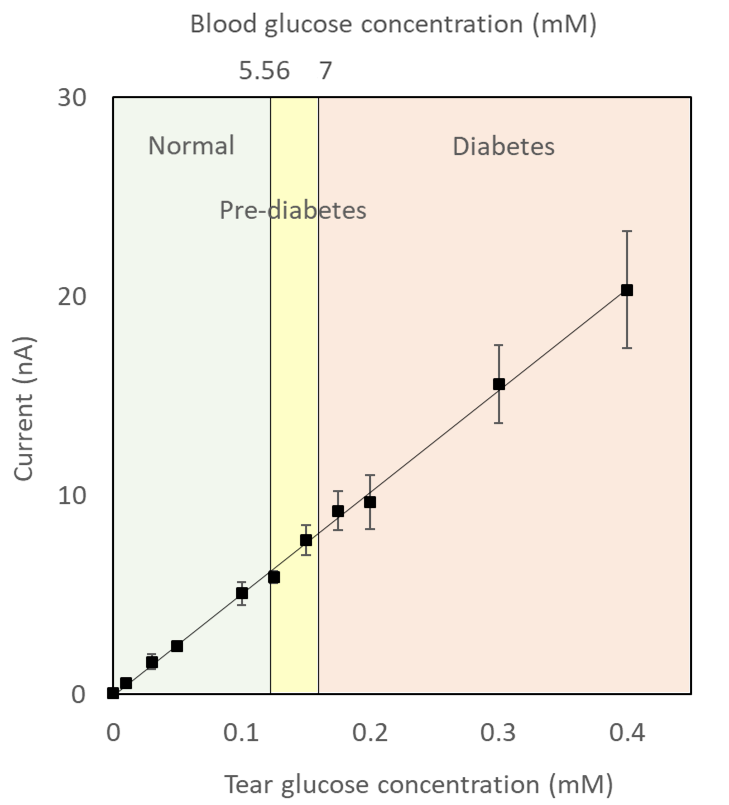
**

**Supplementary Figure 2. Device evaluation at the range of glucose concentration in human tear fluids.** Tear-glucose device developed herein was able to measure the glucose concentrations at the ranges of 0.03 – 0.12 mM, 0.12 – 0.16 mM, and 0.16 mM to higher, which were expected to be present in human tear fluids under the normal, pre-diabetic and diabetic conditions, respectively [^3^](#_ENREF_3)^,^[^4^](#_ENREF_4).

**References**

1 Chu, M. X. *et al.* Soft contact lens biosensor for in situ monitoring of tear glucose as non-invasive blood sugar assessment. *Talanta* **83**, 960-965, doi:10.1016/j.talanta.2010.10.055 (2011).

2 La Belle, J. T. *et al.* Self-monitoring of tear glucose: the development of a tear based glucose sensor as an alternative to self-monitoring of blood glucose. *Chem Commun* **52**, 9197-9204, doi:10.1039/c6cc03609k (2016).

3 Lane, J. D., Krumholz, D. M., Sack, R. A. & Morris, C. Tear glucose dynamics in diabetes mellitus. *Curr Eye Res* **31**, 895-901 (2006).

4 Unwin, N., Shaw, J., Zimmet, P. & Alberti, K. G. M. M. Impaired glucose tolerance and impaired fasting glycaemia: the current status on definition and intervention. *Diabetic Med* **19**, 708-723 (2002).
